# Flow Cytometry-Based Monitoring of Microbial Dynamics During Grape Must Fermentation Under Different Inoculation Strategies

**DOI:** 10.3390/ijms27031414

**Published:** 2026-01-30

**Authors:** Federico Sizzano, Valentina Bianconi, Eddy Dorsaz, Antoine Boilley, Hélène Berthoud, Nadine Bridy, Laurent Amiet, Gilles Bourdin

**Affiliations:** 1 Oenology Research Group, Division of Plant Production Systems, Agroscope, 1260 Nyon, Switzerland; 2 Viticulture and Wine Office, Wallis State Administration, 1950 Sion, Switzerland; 3 Ferments Research Group, Division of Development of Analytical Methods, Agroscope, 3003 Liebefeld, Switzerland

**Keywords:** fermentation, flow cytometry, microbial communities

## Abstract

We applied flow cytometry (FCM) to monitor microbial dynamics during grape must fermentation at the winery scale. Experiments were performed on Pinot Noir grapes using three distinct winemaking protocols: inoculation with active dried yeast, *Pied-de-Cuve*, and spontaneous fermentation. FCM enabled the assessment of yeast viability and metabolic activity, as well as the detection and monitoring of viable bacterial populations during alcoholic fermentation. Amplicon-based DNA sequencing was performed to characterize the associated microbial communities and evaluate protocol-specific effects. Trends identified by amplicon sequencing were partially mirrored by patterns observed in unsupervised FCM analysis. Overall, our results indicate that FCM is a practical tool for monitoring microbial dynamics during fermentation, providing near–real-time information that can support monitoring strategies and risk management in winemaking.

## 1. Introduction

Alcoholic fermentation (AF) is a complex biochemical process in which the dynamics of the microbial community exert a decisive influence on wine chemical composition and sensory expression [1,2]. For this reason, accurate microbiological monitoring is essential, as it allows for the assessment of yeast viability and fermentative performance as well as early detection of spoilage yeasts or bacteria that may compromise the aromatic integrity of the final product [3]. Traditional approaches—such as culture-based assays, microscopic examination, and, more recently, molecular techniques—have greatly contributed to our understanding of fermentative ecosystems [4]. However, these methodologies present important limitations. Culture-dependent assays require long incubation periods and often underestimate viable but non-culturable (VBNC) cells [5]. Microscopy is inherently prone to counting variability and morphological misinterpretation [6], while molecular assays, although highly sensitive and specific, are time-consuming and require specialized expertise and infrastructure, limiting their applicability for real-time monitoring [7].

Flow cytometry (FCM), a gold-standard method in the immunological field, has therefore attracted increasing interest in enological microbiology due to its ability to provide rapid, high-resolution measurements at the single-cell level [8]. Using fluorescent probes that provide information on cell viability, membrane integrity and metabolic activity, FCM yields quantitative and qualitative information that complements conventional monitoring methods [9]. Several studies have demonstrated the usefulness of FCM for tracking AF involving *Saccharomyces* and non-*Saccharomyces* yeasts, as well as malolactic fermentation performed by *Oenococcus oeni* [10,11]. The relevance of this technique has also been formally recognized in recent recommendations by the International Organization of Vine and Wine, which encourages its adoption for microbiological monitoring in winemaking [see https://www.oiv.int/node/4089; accessed on 30 November 2025]. Nevertheless, the use of FCM as an operational monitoring tool under real winery-scale conditions remains limited, especially in spontaneous fermentation (Spo) conditions [12]. A key advantage of FCM lies in its short turnaround time, typically around 20 min, including sample preparation, staining, and data acquisition. This may allow for the rapid identification of stress conditions, shifts in population structure, or early signs of contamination, thus facilitating timely intervention in cellar. Such rapidity is particularly valuable in Spo, in which the absence of active dry yeast (ADY) inoculation results in a process that relies entirely on indigenous microbiota [13]. Although Spo fermentations can enhance aromatic complexity and contribute to varietal typicity, they are also characterized by high variability and increased susceptibility to spoilage organisms [14]. Early fermentation stages are frequently dominated by *non-Saccharomyces* genera such as *Hanseniaspora*, *Candida*, or *Metschnikowia*, whose metabolic activities may strongly influence fermentation kinetics and the production of volatile compounds [15]. In parallel, lactic and acetic acid bacteria may proliferate if temperature, nutrient availability, or oxygen exposure are not adequately controlled, potentially leading to elevated volatile acidity, biogenic amine accumulation, or even stuck fermentation [16]. Continuous, high-resolution monitoring is therefore essential to maintain control over the fermentation trajectory and enable timely corrective interventions [17].

In this study, we extended the application of FCM to Spo, as well as to classic inoculations with ADY and *Pied-de Cuve* (PdC). By comparing microbial trajectories across these fermentation modes, we sought to characterize the associated population dynamics and to assess whether FCM can provide rapid and functionally relevant information on fermentation trajectories, supporting timely operational decisions during winemaking at the cellar level.

## 2. Results

### 2.1. Fermentation Kinetics Reflect the Different Yeast Inoculation Strategies

**Figure 1:**
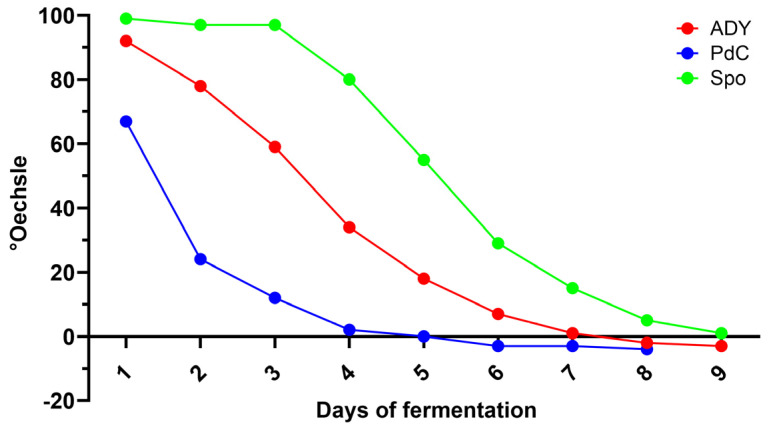
Sugar consumption as measured by densitometry across the three different protocols: ADY: Active Dried Yeast; PdC: *Pied-de-Cuve*; Spo: spontaneous fermentation.

**Figure 2:**
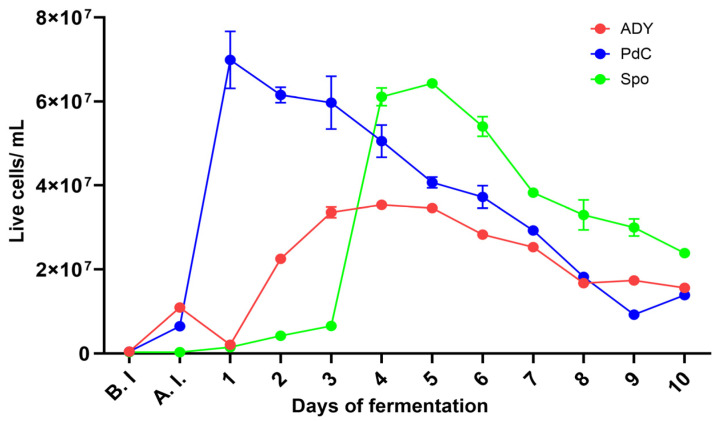
The dynamics of growing cells, as measured by FCM, across the three fermentation protocols: ADY: Active Dried Yeast; PdC: *Pied-de-cuve*; Spo: spontaneous fermentation.

**Figure 3:**
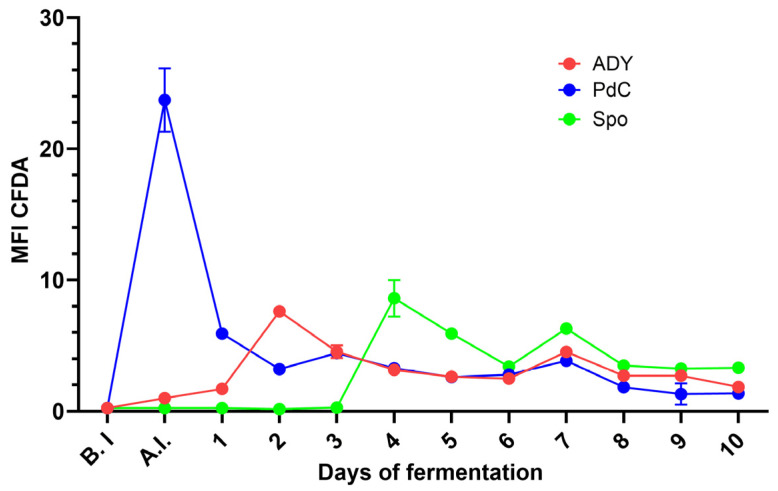
Yeast metabolic activity, as measured by CFDA fluorescence under the three fermentation protocols. ADY: Active Dried Yeast; PdC: *Pied-de-Cuve*; Spo: spontaneous fermentation.

Marked differences in the progression of AF were observed among the three inoculation strategies. PdC condition exhibited the fastest sugar consumption from the early stages of fermentation, reaching negative densitometry values within 5 days. In contrast, ADY showed a short latency period before accelerating its fermentative activity, with sugar levels decreasing and approaching negative densitometry values between days 7 and 8. Spo displayed the slowest kinetics, characterized by an extended initial plateau and a delayed onset of rapid sugar conversion (Figure 1). In terms of viable and active yeast, PdC reached its maximum viable cell concentration one day after inoculation, exceeding 6–7 × 10^7^ cells/mL. The ADY condition showed a more classical growth curve, with an initial low concentration of viable cells followed by a rapid expansion between days 1 and 4, peaking around 3–4 × 10^7^ cells/mL. The Spo condition began with minimal viable yeast counts but exhibited substantial growth starting around days 2–3, eventually reaching population sizes comparable to PdC (Figure 2). Measurements of metabolic activity using 5-carboxy-fluorescein diacetate-acetoxymethyl ester (CFDA-AM) median fluorescence intensity (MFI) detected the physiological states of the fermenting populations. The PdC condition displayed an early and pronounced MFI peak at inoculation, indicative of highly active and responsive cells entering fermentation. This peak was followed by a rapid stabilization at lower intensity values. The ADY and Spo conditions generated more variable MFI patterns, with moderate and fluctuating activity levels throughout the process. Spo exhibited a delayed increase in MFI around day 4, suggesting a progressive physiological transition to active fermentation (Figure 3). A phase-specific area under the curve (AUC) analysis was performed on longitudinal cell concentration and CFDA fluorescence data (days 0–3, 4–6, and 7–10 after inoculation). PdC showed the highest early-phase AUC values for both cell concentration and CFDA, indicating rapid establishment and activity, whereas Spo displayed lower early values followed by higher mid- and late-phase AUCs, consistent with delayed but sustained dynamics. ADY exhibited intermediate profiles across phases. Detailed AUC values and confidence intervals are reported in Supplementary Figure S2.

### 2.2. Live Bacterial Populations Across Fermentation Protocols

**Figure 4:**
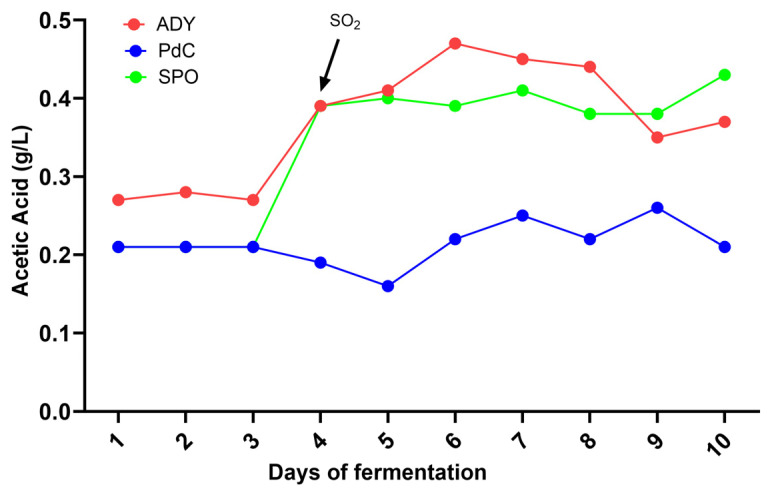
Follow-up of acetic acid. The arrows indicate the addition of SO_2_ (10 mg/L) to the three conditions. ADY: Active Dried Yeast; PdC: *Pied-de-Cuve*; Spo: spontaneous fermentation.

**Figure 5:**
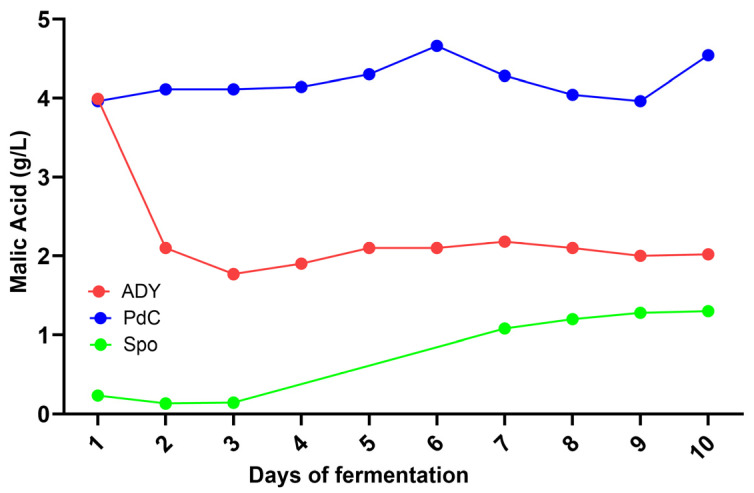
Follow-up of malic acid. ADY: Active Dried Yeast; PdC: *Pied-de-Cuve*; Spo: spontaneous fermentation.

**Figure 6:**
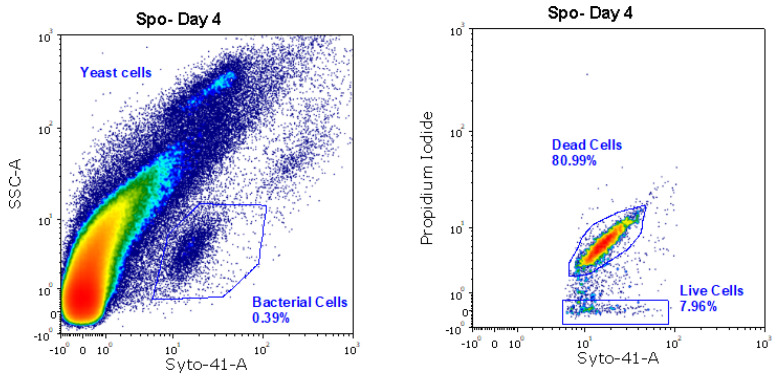
Bacteria quantification on day 4 by FCM. Left dot plot: bacteria population (low-SSC). Right-dot plot: viability of bacteria population. Spo: spontaneous fermentation.

**Figure 7:**
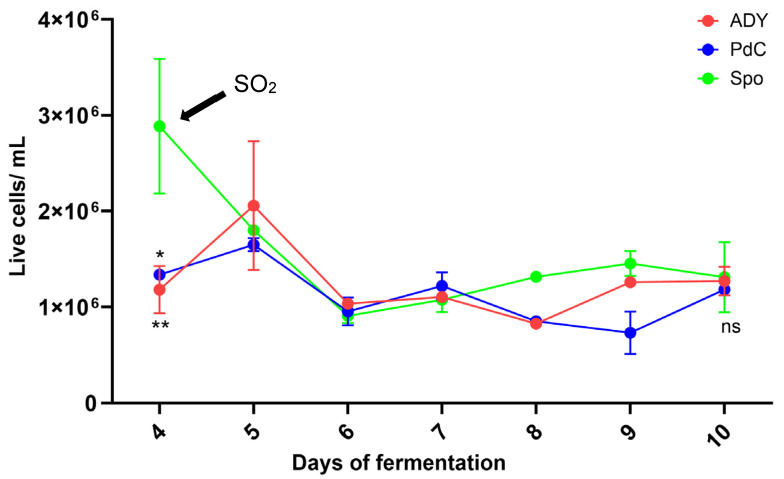
Monitoring of bacterial population throughout the fermentation, after the addition of SO_2_. ADY: Active Dried Yeast; PdC: *Pied-de-Cuve*; Spo: spontaneous fermentation. One way-ANOVA *: *p* < 0.05 Spo vs. PdC; **: *p* < 0.01 Spo vs. ADY at day 4: ns: no significant differences at day 10.

On the fourth day of fermentation, we observed an increase in acetic acid in the ADY and Spo conditions (Figure 4). Interestingly, under such conditions, a decrease in malic acid concentration was observed from day 1 (Figure 5). FCM analysis indicated a FSC^low^/SSC^low^/Syto-41^pos^/propidium iodide (PI) ^pos/neg^ population at this time point, consistent with the presence of bacterial cells (Figure 6). Notably, the Spo condition displayed a significantly higher abundance of viable bacteria than the ADY and PdC conditions (Spo: 2.9 × 10^6^ cells/mL; ADY: 1.1 × 10^6^ cells/mL; PdC: 1.3 × 10^6^ cells/mL; Figure 7). Given the rise in acetic acid and the evidence of live bacteria, all fermentations were treated with 10 mg/L of sulfur dioxide (SO_2_) at this time point as a precautionary measure to inhibit further bacterial growth. Following SO_2_ addition, we initiated a systematic bacterial follow-up alongside yeast monitoring. FCM showed that the bacterial population stabilized rapidly in all three conditions. Most notably, the Spo condition—which initially exhibited the highest bacterial load—displayed a progressive decrease in viable bacterial cells after treatment (Figure 7). This stabilization pattern persisted in the subsequent stages of fermentation and paralleled the attenuation of acetic acid accumulation observed after SO_2_ addition.

### 2.3. Molecular Analysis Shows Complexity of Microbial Consortia and Is Partially Mirrored by Unbiased Flow Cytometry Analysis

DNA sequencing revealed differences in microbial community composition among the three fermentation strategies at different fermentation phases.

**Figure 8:**
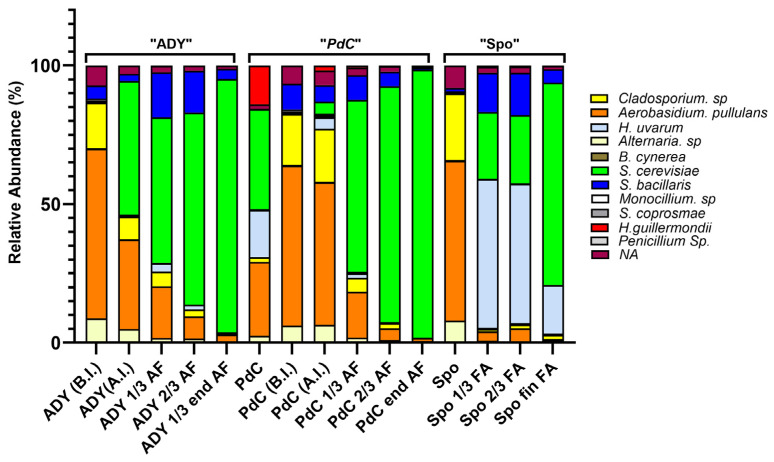
Relative yeast DNA abundance throughout the fermentation in the three conditions. ADY (B.I): Active Dried Yeast must before the inoculation of *S. cerevisiae*; ADY (A.I.): Active Dried Yeast must after the inoculation of *S. cerevisiae*; ADY 1/3, 2/3, end AF: Active Dried Yeast at 1/3, 2/3, and at the end of AF; PdC: *Pied-de-Cuve*; PdC (B.I.): condition before the inoculation of the *Pied-de-Cuve*; PdC (A.I.): condition after the inoculation of the *Pied-de-Cuve*; PdC 1/3, 2/3, end AF: *Pied-de-Cuve* at 1/3, 2/3, and at the end of AF; Spo: spontaneous fermentation tank after grape crushing; Spo 1/3, 2/3, end AF: spontaneous fermentation at 1/3, 2/3, and at the end of AF.

Before inoculation, all conditions contained a community consisting mainly of yeast and fungi, such as *S. bacillaris*, *A. pullulans* and *Cladosporium* sp. After inoculation, *S. cerevisiae* became the most prominent species, especially in the ADY and PdC conditions, from 1/3 of AF onwards. The Spo condition exhibited the greatest complexity, with *H. uvarum* and *S. cerevisiae* present until the end of fermentation. *S. bacillaris* was found at low levels in all conditions (see Figure 8).

**Figure 9:**
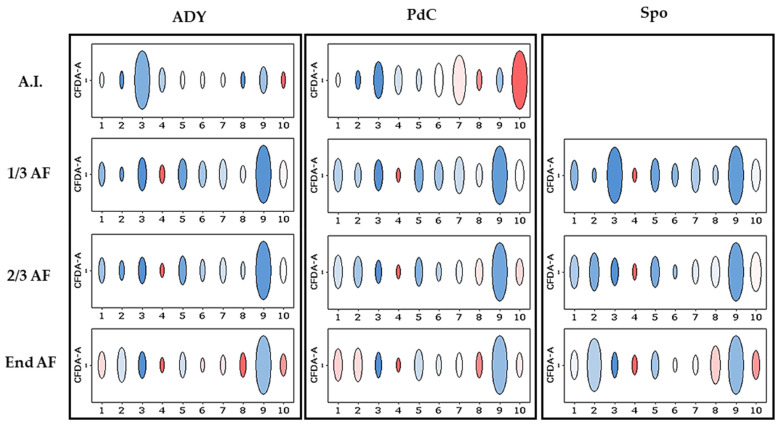
Relative abundance of metaclusters throughout fermentation in the three conditions. Circle size reflects the percentage of cells within each metacluster, as defined by FlowSOM (clusters 1–10). ADY (A.I.): Active Dried Yeast must after the inoculation of *S. cerevisiae*; ADY 1/3, 2/3, end AF: Active Dried Yeast at 1/3, 2/3, and at the end of AF; PdC: *Pied-de-Cuve*; PdC (A.I.): condition after the inoculation of the *Pied-de-Cuve*; PdC 1/3, 2/3, end AF: *Pied-de-Cuve* at 1/3, 2/3, and at the end of AF; Spo: spontaneous fermentation; Spo 1/3, 2/3, end AF: spontaneous fermentation at 1/3, 2/3, and at the end of AF. Spo A.I. contained very few events and was not included in the analysis. The blue-white-red color gradient shows increasing levels of CFDA fluorescence.

We then applied an unsupervised FCM analysis, based on the hypothesis that different yeast populations may exhibit distinct phenotypic patterns in multidimensional space. Figure 9 shows the population structure as defined by the FlowSOM algorithm in the three fermentation conditions after inoculation and at 1/3, 2/3, and end of the AF. The relative abundance of the defined metaclusters varied markedly according to fermentation mode and phase. With the ADY protocol, a single metacluster predominated at all stages (1/3 AF, 2/3 AF, and end of AF), with a reduced representation of the other clusters. Diversity was higher with the PdC protocol, especially under the A.I. condition. However, from 1/3 AF, only one metacluster was predominant. In Spo, at least two predominant metaclusters were represented at 1/3 of AF and at the end of AF.

**Figure 10:**
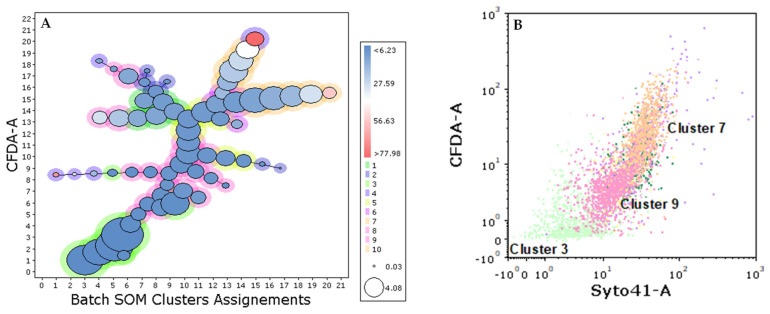
(**A**) Yeast phenotypic structure in the spontaneous fermentation (Spo) condition at one-third of alcoholic fermentation (1/3 AF). Circles represent clusters identified by the FlowSOM algorithm. Circle color (blue–white–red) indicates increasing levels of CFDA fluorescence intensity, while circle size reflects the relative percentage of cells within each cluster. Phenotypically similar clusters are grouped into metaclusters (10 metaclusters in this experiment; see legend). (**B**) Dot plot highlighting the most abundant metaclusters detected in the Spo condition at 1/3 AF.

Figure 10 illustrates the population structure of the Spo condition at 1/3 AF, together with the corresponding dot plot highlighting the most abundant metaclusters identified by FlowSOM.

**Figure 11:**
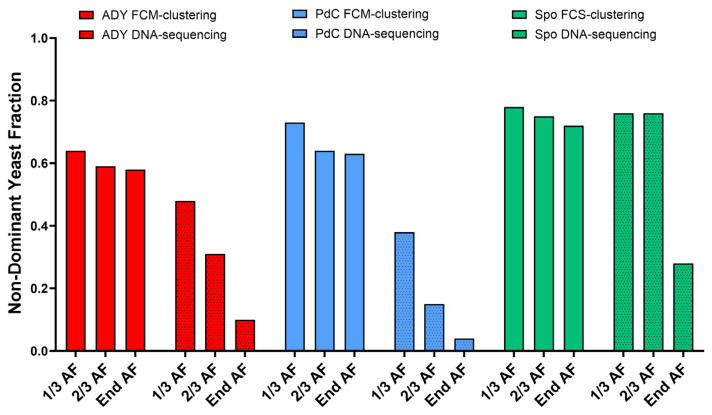
Non-dominant fraction of microbial community for DNA-sequencing and FCM data. The non-dominant fraction was defined as 1 minus the relative abundance of the dominant taxonomic component (*S. cerevisiae* in sequencing) or phenotypic metacluster (Cluster 9 in FCM) and was used as a quantitative descriptor of community convergence. ADY: Active Dried Yeast; *PdC*: Pied-de-Cuve; Spo: spontaneous fermentation.

We reasoned that heterogeneous fermentations would have less contribution from the dominant species (i.e., *S. cerevisiae*). Thus, to provide a quantitative comparison between sequencing- and FCM-derived descriptions of community structure, we calculated the fraction of non-dominant yeasts independently from sequencing data and from FlowSOM-derived metacluster percentages. This metric reflects the cumulative contribution of all components other than the dominant one and was used as a proxy for community convergence during fermentation. Across all sampling points, Spo consistently exhibited higher non-dominant fractions compared to inoculated conditions. This pattern was particularly evident in the amplicon sequencing data, while FCM captured the same trend with lower contrast (Figure 11).

### 2.4. No Differences in Wine Chemical Parameters or Sensory Evaluation Among the Wines Produced with the Different Inoculation Strategies

**Figure 12:**
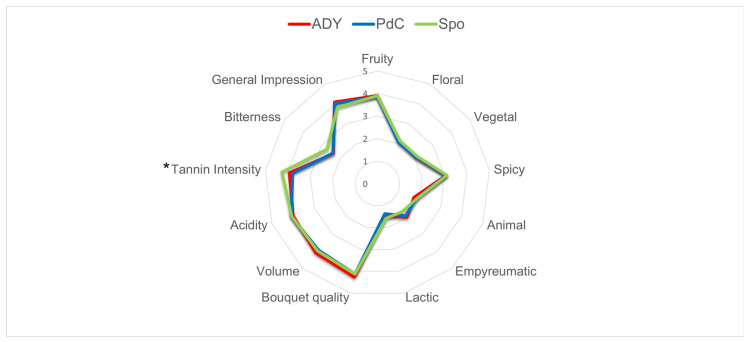
Radar graph showing the sensory profile of the ADY, PdC, and Spo wines. Asterisk indicates significant difference in tannin intensity between PdC and Spo (*p* < 0.05).

Biochemical analyses performed by FTIR (Fourier Transform InfraRed Spectroscopy) and sequential enzymatic assays did not reveal major differences among wines produced using the different inoculation methods (Table 1). Although a formal statistical comparison was not possible, PdC wines showed a lower ethanol content, while Spo wines exhibited higher levels of lactic acid and glycerol compared with both PdC and ADY. Sensory analysis conducted by an expert panel (16 panelists) revealed no significant differences among the three wines, except for a significantly higher tannin intensity (*p* < 0.05) in Spo wine compared with PdC (Figure 12).

## 3. Discussion

In the present study, we characterized the microbiological and physiological trajectories of AF under active ADY inoculation, PdC, and Spo, revealing distinct modality-dependent patterns at the winery scale through the combined use of oenological analyses, FCM, and amplicon-based sequencing. The differences observed in sugar consumption and yeast population dynamics highlight the impact of the inoculation strategy on the onset and progression of AF. PdC condition showed the fastest decrease in densitometric values and rapidly reached high viable cell counts after inoculation, consistent with the transfer of a pre-adapted, actively fermenting consortium. ADY, in contrast, displayed a short lag before entering the exponential phase, reflecting the time required for rehydrated yeast to acclimate to must conditions. Spo fermentation progressed more slowly, with an extended initial plateau in densitometry and a delayed increase in viable cells. This pattern is consistent with the fact that without an exogenous starter agent, the indigenous microbiota must reorganize in succession to establish a fermentative community [18]. The CFDA-based metabolic activity profiles reinforce this view: PdC exhibited a marked metabolic peak at inoculation, whereas ADY and especially Spo showed more gradual and fluctuating activation, with Spo only reaching higher CFDA values after several days. These observations show that FCM can capture not only quantitative changes in yeast populations but also qualitative transitions in the physiological state that precede or accompany shifts in the fermentation rate.

The parallel changes in acetic and malic acid observed during the early stages of fermentation highlight the relevance of microbiological dynamics, particularly in fermentation modalities that rely on indigenous microbiota and in musts not treated with sulfites. A decrease in malic acid was observed from day 1, and on day 4, both ADY and Spo conditions exhibited an increase in acetic acid. FCM enabled the detection of viable bacterial cells during alcoholic fermentation. This observation prompted the application of 10 mg/L SO_2_ at this time point as a precautionary intervention to limit potential bacterial contributions to acetic acid formation. Following SO_2_ addition, a progressive decline in viable bacterial cells was observed in the Spo condition, accompanied by an attenuation of acetic acid accumulation.

The temporal association between bacterial viability, early malic acid decrease, and acetic acid accumulation suggests a potential contribution of bacterial activity during the initial fermentation stages in the ADY and Spo conditions. The presence of acetic acid in both ADY and Spo, coupled with the fact that *H. uvarum* (known to produce acetic acid) was mainly present in Spo, is consistent with a bacterial contribution to acetic acid accumulation, although a contribution from yeast metabolism cannot be excluded [19]. The absence of malic acid consumption and acetic acid production in PdC is consistent with the hypothesis that the highly active yeast population in the inoculum prevented the bacterial population from becoming functional, despite their presence. However, under the staining conditions used in this study, the phenotypic resolution of the FCM setup was insufficient to reliably distinguish between different bacterial groups. As a consequence, a more specific attribution of the observed effects to particular bacterial populations was not possible. This limitation highlights the need for complementary approaches to better characterize bacterial populations and to guide more targeted interventions during fermentation. Future studies could benefit from the integration of species-specific probes (e.g., antibodies available for *Oenococcus oeni*) with FCM strategies that exploit structural differences in the cell walls of lactic and acetic acid bacteria (e.g., fluorescent Gram staining with hexidium iodide) [20,21]. Notably, the increase in malic acid observed after day 4 in the Spo condition was unexpected. In the context of a metabolically heterogeneous and dynamically evolving microbial ecosystem, such a trend could be associated with alterations in central carbon metabolism. Although a yeast-mediated redistribution of carbon toward anaplerotic, TCA-related intermediates represents one possible explanation [22], additional data are required to support this hypothesis and to clarify the underlying metabolic mechanisms.

Amplicon-based DNA sequencing of the yeast community in our samples provided a complementary taxonomic perspective on fermentation processes. Before inoculation, all conditions shared a community including *S. bacillaris*, *A. pullulans*, and *Cladosporium* spp., likely reflecting the microbiota present on the grapes and in the must. After the addition of ADY or PdC, *S. cerevisiae* became the dominant species from one-third of AF onward, confirming the strong selective pressure exerted by these inoculation strategies. Under the Spo protocol, in contrast, the community remained more complex for a longer period, with *H. uvarum* and *S. cerevisiae* co-detected until the end of AF. *S. bacillaris* persisted at low relative abundance across all fermentation conditions. This observation is consistent with our previous data from independent fermentations [23] and suggests that *S. bacillaris* may persist by exploiting metabolic niches that are not directly competed for by *S. cerevisiae*. Together, these results indicate that the Spo condition maintains higher yeast diversity over time and that AF may proceed within a mixed microbial community rather than within a population dominated exclusively by *S. cerevisiae* [24].

Unsupervised FCM analysis using FlowSOM enabled the comparison of phenotypic profiles across fermentation strategies and over time, based on variations in the relative abundance of shared metaclusters. Although the same set of metaclusters was identified in all conditions, their relative representation differed according to inoculation strategy and fermentation stage. In the ADY condition, a single metacluster rapidly became dominant, consistent with the reduced phenotypic complexity typically observed in fermentations dominated by a single yeast population such as *S. cerevisiae*. In PdC, several metaclusters were initially detected, followed by a progressive convergence toward a dominant phenotype, whereas Spo maintained a more heterogeneous profile for a longer portion of fermentation. This pattern is in line with the higher microbial complexity observed in Spo by DNA sequencing, suggesting an association between yeast diversity and phenotypic heterogeneity. Consistently, a comparison of the non-dominant fraction derived independently from sequencing and FCM indicated that Spo retained a higher contribution of non-dominant yeasts, compared to ADY and PdC, throughout fermentation, reflecting delayed community convergence. However, under the staining conditions employed, direct assignment of metaclusters to specific yeast species was not possible. In this context, the development of yeast-specific antibodies or molecular probes—currently limited in oenological applications [25]—would substantially improve the taxonomic resolution of FCM analyses. When combined with functional probes, these tools could make FCM a powerful approach for the real-time characterization of microbial communities, thereby enabling more precise management of fermentation trajectories. Even under the current methodological constraints, the number and persistence of dominant metaclusters may represent a useful and rapid operational fingerprint of fermentation dynamics, with delayed convergence—such as that observed with the Spo protocol—reflecting greater functional heterogeneity at the population level.

Sensory analysis revealed no significant differences among the three wines; however, the Spo condition showed higher perceived tannin intensity compared to the ADY and PdC. The potential involvement of non-*Saccharomyces* yeasts, including *H. uvarum*, in modulating tannin-related mouthfeel is noteworthy, although previous studies in Aglianico winemaking have reported contrasting effects, underscoring the strong context dependence of such outcomes [26]. The overall sensory appreciation did not differ among wines, and no major sensory defects were detected, particularly in the Spo condition. This outcome may partly reflect the close analytical and microbiological monitoring applied throughout fermentation. From a winery perspective, the present work shows that FCM can be integrated into the monitoring of both inoculated and Spo fermentations without disrupting cellar routines. In inoculated fermentations (ADY and PdC), FCM can rapidly confirm starter establishment, yeast physiological robustness, and the absence (or containment) of bacterial proliferation. In Spo, where microbial diversity is intentionally preserved, FCM offers a rapid way to follow the trajectory of complex populations and to identify critical phases in which bacterial activity or changes in yeast physiology may warrant intervention. In complex fermentation, FCM captures shifts in population dominance that cannot be readily accessed by traditional methods. However, conventional DNA-based methods are still essential for species-level identification. Further work, including the replication of this approach across vintages, grape varieties, and cellar environments, will be essential to validate the generality of the patterns described here. However, microbial dynamics may vary between wineries as a result of differences in grape composition, indigenous microbiota, and processing conditions. Nonetheless, the present results already support the view that FCM, used in combination with classical and molecular tools, can substantially enhance our ability to manage microbiological risks while preserving the diversity and identity of different vinification practices.

## 4. Materials and Methods

### 4.1. Cellar-Scale Experiment

Pinot Noir grapes (about 800 kg) from the Grand-Brulé domain of Leytron (Wallis Canton, Switzerland) were harvested and processed on 20 September 2024. After destemming and crushing, the grapes were divided into three tanks (240 kg/tank), corresponding to the condition Active Dried Yeast (ADY), *Pied-de-Cuve* (PdC) and spontaneous fermentation (Spo). The ADY condition was inoculated with Lalvin RC212 (Lallemand, Montreal, QC, Canada) at a concentration of 20 g/hL. For the PdC condition, the starter was prepared by harvesting and processing 30 kg of grapes one week before the official harvest. Ten mg/L of SO_2_ was added to favor fermentative yeasts. The spontaneously fermenting must (with a densitometric value of 40° Oechsle) was added to the PdC tank at an approximate ratio of 1:10, concomitant with ADY inoculation. Spo fermentation was left not inoculated. The tanks were kept closed and were open only once per day to manually mix the grapes and sample the juice in fermentation. To compensate for the low content of yeast assimilable nitrogen in the musts, which was approximately 120 mg/L, 13 g/hL of diammonium phosphate, resulting in 30 mg/L of yeast assimilable nitrogen, was added to each tank on day 1.

AF was carried out at 22–25 °C and monitored daily by densitometry using DMA35 (Anton Paar, Graz, Austria). The AF was considered as finished after 5–7 consecutive days of negative densitometric values and detection of residual sugar (<1 g/L). The grapes were then pressed using an EPC 25 press (Sutter, Villigen, Switzerland) and racking was carried out after 3 days. Residual malic acid was consumed by malolactic fermentation (MLF) after inoculation with *Oenococcus oeni* (Vitilactic F, Martin Vialatte, Magenta, France). At the end of fermentation (malic acid levels < 0.1 g/L), the wines were chemically stabilized with sulfites (40 mg/L) and later physically stabilized by cooling at 1 °C for 1 month. Bottling (March 2024) was preceded by an additional cartridge filtration step at 0.65 and 0.45 μM. Bottles were stored under controlled conditions before analysis (10–12 °C in the dark).

### 4.2. Flow Cytometry (FCM)

For FCM analysis, samples of fermenting red must were filtered using 100 μm cell strainers (Greiner, Krensmünster, Austria), diluted 1:100 in Phosphate Buffer Saline (PBS). Cells were stained with 5-carboxy-fluorescein diacetate-acetoxymethyl ester (CFDA-AM; Thermo Fisher, Waltham, MA, USA) as a general indicator of metabolic activity (final concentration of 0.5 μM), Syto-41 nucleic acid stain (Thermo Fisher) to label total nuleic-acids containing events (1 μM final concentration), and propidium iodide (PI, Sigma-Aldrich, St. Louis, MO, USA) at 0.5 μg/mL to identify dead cells. After incubation for 15 min at room temperature, the sample was collected using a volumetric MACSQuant 10 analyzer (Miltenyi Biotec, Bergisch Gladbach, Germany). Offline analysis of the FCM files was performed using Flow Logic software (Version 8.7, Inivai Technologies, Mentone, Australia). Supplementary Figure S1 illustrates a general gating strategy. The parameters analyzed included the cell count (expressed as live cells/mL) and the relative fluorescence intensity of CFDA (expressed as median fluorescence intensity [MFI]), a general indicator of the metabolic activity of the cell. Unsupervised analysis was carried out using FCS Express v 7.28.0035 (Dotmatics, Boston, MA, USA) implementing the FlowSOM algorithm. FlowSOM applies a self-organizing map (SOM) to group cells based on phenotypic similarity, followed by hierarchical consensus clustering to define metaclusters (10 in this experiment). Parameters included Syto-41 and CFDA fluorescence, forward and side scatter (hyperlog-transformed), an 8 × 8 SOM grid, and downsampling to 4000 events within the live-cell gate.

### 4.3. Amplicon-Based Sequencing After Cellar-Scale Fermentation

DNA extraction and amplicon sequencing were performed as previously described by Sizzano et al. [23], with minor adaptations. Briefly, must samples (50 mL) were centrifuged, and the resulting pellets were stored at −20 °C until processing. After washing in saline peptone water, the cells were recovered by centrifugation and subjected to mechanical disruption using zirconia beads in a Bead Ruptor system (OMNI International, Kennesaw, GA, USA). Cell lysis was completed by CTAB treatment, followed by chloroform–isoamyl alcohol extraction. Genomic DNA was subsequently purified using the EZ1 DNA Tissue Kit (Qiagen, Hilden, Germany) and quantified by NanoDrop (Thermo Fisher) spectrophotometry.

We targeted a portion of the translation elongation factor 1-α (TEF1α) gene because general ITS2 metabarcoding can be biased by rDNA copy-number variation, affecting both taxonomic resolution and relative abundance estimates. Low-copy protein-coding markers such as TEF1α improve species-level discrimination and reduce copy-number-driven bias in yeasts [27].

TEF1α amplicon libraries were prepared using a two-step unidirectional fusion PCR approach (Thermo Fisher). The first PCR was performed using universal fungal primers, followed by purification and a second PCR to add sequencing adapters and barcodes. Amplicon quality and concentration were assessed using an Agilent 2100 Bioanalyzer (Agilent Technologies, Santa Clara, CA, USA), after which libraries were pooled equimolarly, purified, and diluted to a final concentration of 50 pM. Sequencing was carried out on an Ion Torrent platform using an Ion 530 chip (Thermo Fisher). Raw sequences were quality-filtered and processed in DADA2 to generate amplicon sequence variants (ASVs), and taxonomic assignment was performed using BLAST (Version 2.16.0)-based annotation.

### 4.4. Must and Wine Analysis

#### 4.4.1. Wine Analysis by Fourier Transform Infrared (FT-IR) and Enzymatic Methods

Samples collected during AF or at the end of the entire winemaking process (bottled wine) were analyzed by enzymatic methods (Biosystems, Barcelona, Spain) or FTIR Wine Scan (Foss, Hillerod, Denmark). The main parameter measures included malic, lactic, tartaric or acetic acids, sugars, ethanol, glycerol, or anthocyanins.

#### 4.4.2. Sensory Analysis

A few weeks after bottling, a sensory profile of the cellar-scale experimental wines was performed by a trained panel of 16 tasters from Agroscope using Redjade (version 7.4.3, Redjade Sensory Solutions, Martinez, CA, USA). The tasters assessed the intensity of different criteria on a scale from 1 (low/poor) to 7 (high/excellent). The three modalities were tasted comparatively; 50 mL of wine was served at 17 ± 1 °C in transparent INAO glasses, anonymized by a three-digit code, and presented in different orders to the panelists.

### 4.5. Statistical Analysis

Densitometric data were used descriptively to illustrate temporal trends in sugar consumption. Daily measurements for live cells and CFDA represent the mean of three independent subsamples collected from each fermentation tank. An area under curve (AUC) analysis was performed to analyze the kinetics of fermentation and cell metabolic activity, dividing fermentation into three sub-periods: days 0–3; days 4–6, and days 7–10. The difference in cell concentration at individual time points between fermentation protocols was assessed using a one-way analysis of variance (ANOVA), while a mixed-effect model (with Tukey’s post-tests) was applied to evaluate sensory differences between wines. GraphPad Prism 10 (Dotmatics, Boston, MA, USA) was used for all calculations. 

## Figures and Tables

**Table 1:** Oenological parameters of bottled wines produced under the three conditions.

	pH	Titrable Acidity (g/L)	Tartaric Acid (g/L)	Malic Acid (g/L)	Acetic Acid (g/L)	Lactic Acid (g/L)	Glycerol (g/L)	Ethanol (%)	Residual Sugars (g/L)	Antocyanins (mg/L)
**ADY**	3.95	4.1	<1.0	0.3	0.38	2.54	8.6	13.0	<1	285
**PdC**	3.92	4.1	1.1	<0.1	0.30	2.45	8.6	11.4	<1	273
**Spo**	3.92	4.5	<1.0	<0.1	0.35	3.35	9.3	12.6	<1	284

## Data Availability

Data are contained within the article and the Supplementary Materials.

## Supplementary Materials

The following supporting information can be downloaded at: https://www.mdpi.com/article/10.3390/ijms27031414/s1.

## Abbreviations

The following abbreviations are used in this manuscript:

FCM	Flow Cytometry
AF	Alcoholic Fermentation
ADY	Active Dry Yeast
PdC	*Pied-de-Cuve*
Spo	Spontaneous Fermentation
CFDA	5-Carboxyfluorescein Diacetate, Acetoxymethyl Ester
PI	Propidium Iodide
MFI	Median Fluorescence Intensity
SOM	Self-Organizing Map
