# Supplementary material for: Flow Cytometry-Based Monitoring of Microbial Dynamics During Grape Must Fermentation Under Different Inoculation Strategies
Source: Int J Mol Sci. 2026 Jan 30;27(3):1414. doi: 10.3390/ijms27031414 (PMC12898849; doi:10.3390/ijms27031414)
Supplement: Supplementary file 1 [file ijms-27-01414-s001.zip › Figures S1 and S2.pdf]

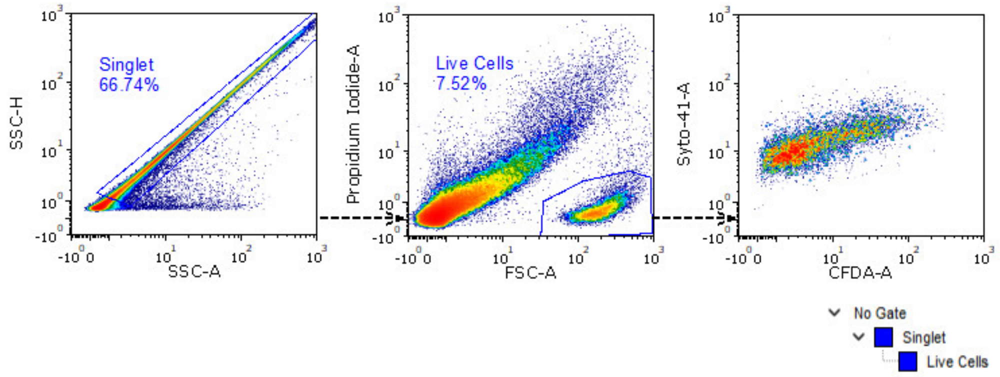

Figure S1. Exemplative FCM gating strategy of yeast population during fermentation.

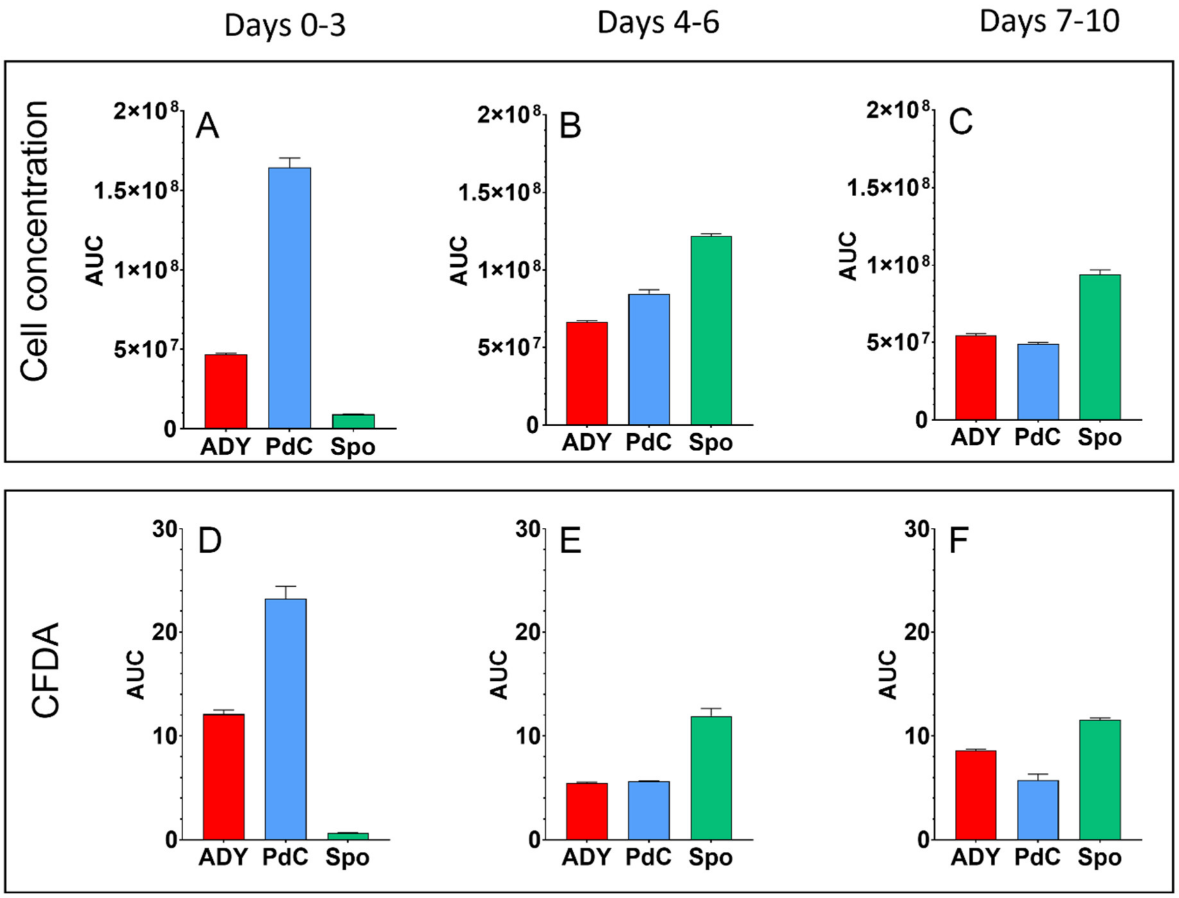

Figure S2. Area under the curve (AUC) analysis for cell concentration and CFDA fluorescence values. A, B, C: AUC for cell concentration; D, E, F: AUC for CFDA. Fermentation was divided into three intervals after inoculation: day 0-3, days 4–6, and days 7–10. **A**: $AUC_{ADY} = 4.7 \times 10^7$; confidence interval (CI) = $[4.5 \times 10^7, 4.8 \times 10^7]$; $AUC_{PdC} = 1.6 \times 10^8$; CI = $[1.56 \times 10^8, 1.73 \times 10^8]$; $AUC_{Spo} = 9.1 \times 10^6$; CI = $[8.6 \times 10^6, 9.6 \times 10^6]$. **B**: $AUC_{ADY} = 6.6 \times 10^7$; CI = $[6.5 \times 10^7, 6.8 \times 10^7]$; $AUC_{PdC} = 8.4 \times 10^7$; CI= $[7.9 \times 10^7, 8.9 \times 10^7]$; $AUC_{Spo} = 1.2 \times 10^8$; CI = $[11.8 \times 10^8, 12.5 \times 10^8]$. **C**: $AUC_{ADY} = 5.4 x 10^7$; CI = $[5.2 x 10^7, 5.6 x 10^7]$; $AUC_{PdC} = 4.9 x 10^7$; CI = $[4.6 x 10^7, 5.1 x 10^7]$; $AUC_{Spo} = 9.4 x 10^7$; CI = $[8.8 x 10^7, 9.9 x 10^7]$. **D:** $AUC_{ADY} = 12.1$; CI = [11.3, 12.9]; $AUC_{PdC} = 23.2$; CI = [20.7, 25.7]; $AUC_{Spo} = 0.66$; CI = [0.59, 0.73]; **E:** $AUC_{ADY} = 5.5$; CI = [5.3, 5.6]; $AUC_{PdC} = 5.6$; CI = [5.5, 5.7]; $AUC_{Spo} = 11.9$; CI = [10.5, 13.3]. **F:** $AUC_{ADY} = 8.6$; CI = [8.3, 8.8]; $AUC_{PdC} = 5.7$; CI = [4.6, 6.9]; $AUC_{Spo} = 11.5$; CI = [11.2, 11.9].
